# Splenic rupture following endoscopic mucosal resection: A case report and literature review

**DOI:** 10.1097/MD.0000000000039846

**Published:** 2024-10-04

**Authors:** Yusong Ye, Rui Yang, Shicheng Peng, Qilang Xiang, Yuexi Chen, Muhan Lü, Weixing Yang

**Affiliations:** a Department of Gastroenterology, The Affiliated Hospital of Southwest Medical University, Luzhou, China; b Nuclear Medicine and Molecular Imaging Key Laboratory of Sichuan Province, Luzhou, China; c School of Graduates, Dalian Medical University, Dalian City, Liaoning Province, China; d Department of Gastroenterology, Chongqing University Three Gorges Hospital, Chongqing, China; e Department of Gastroenterology, The Affiliated Traditional Chinese Medicine Hospital of Southwest Medical University, Luzhou, China.

**Keywords:** case report, colonoscopy, endoscopic mucosal resection, splenic rupture, visceral injury

## Abstract

**Rationale:**

This study aims to highlight the rare but severe complication of splenic rupture following colorectal endoscopic mucosal resection (EMR), advocating for increased vigilance during procedures near the splenic flexure.

**Patient concerns:**

We present a case report of a 66-year-old woman who experienced persistent abdominal pain after undergoing EMR for an adenomatous lesion in the distal transverse colon.

**Diagnoses:**

The diagnosis of splenic rupture was established following her symptoms and clinical evaluation.

**Interventions:**

Active conservative management was implemented after diagnosis.

**Outcomes:**

The patient’s recovery underscores the importance of prompt diagnosis and careful monitoring.

**Lessons:**

Although splenic rupture after EMR is extremely rare, it is a serious and potentially life-threatening complication. When obtaining informed consent, it is important to emphasize not only common complications like bleeding and perforation but also the risk of splenic injury. Physicians should select appropriate instruments and carefully adjust the angle and force of needle insertion, avoiding excessively long needles and vertical insertion. The procedure should be performed gently to minimize the risk of splenic rupture. For lesions near the splenic flexure, if postoperative abdominal pain occurs, regardless of left shoulder pain, splenic rupture should be considered, and a computed tomography scan promptly performed. Postoperatively, physicians should closely monitor vital signs and repeatedly check blood counts and coagulation parameters. Treatment should be tailored to the splenic injury’s extent and the patient’s overall condition, with immediate surgery if necessary. High-risk patients should be regularly followed up and instructed to monitor for physical changes. Endoscopists should remain vigilant during procedures, fully understanding potential complications, and closely monitoring the patient’s condition postoperatively. This vigilance is key to preventing severe complications and ensuring optimal outcomes.

## 1. Introduction

With advancements in endoscopic technology and the integration of artificial intelligence, detection rates of hidden polyps, adenomas, and early colorectal cancer have significantly improved. These advancements have notably enhanced the prevention of colorectal cancer. Endoscopic mucosal resection (EMR), an endoscopic technique developed from polypectomy and mucosal injection methods, is suitable for lesions smaller than 20 mm, as well as flat or laterally spreading tumors.^[1]^ The colon is the most common site for EMR, and over the past decade, EMR has become the fundamental technique for removing colorectal polyps. It effectively and completely removes polyps with a high R0 (complete) resection rate.^[2]^ Large prospective studies have demonstrated that EMR is a safe, effective, and cost-efficient procedure.^[2,3]^

Despite the overall high safety of EMR, there are still risks of complications. Major adverse effects associated with colonic EMR include bleeding, perforation, and post-polypectomy coagulation syndrome. The incidence of post-EMR bleeding ranges from 2% to 24%, particularly common in patients with large right-sided polyps and those receiving antithrombotic therapy. The incidence of perforation is about 1%, with severe cases possibly requiring emergency surgery. The incidence of post-polypectomy coagulation syndrome is 0.003% to 1%, usually with a good prognosis through supportive treatment.^[2]^ However, EMR may also induce some underestimated, life-threatening rare complications. For instance, in 2018, Ohtsuka et al^[4]^ documented a case of retroperitoneal hematoma occurring 11 days post-EMR. To date, there have been no reports of splenic injury post-EMR.

In this report, we describe a patient who presented with abdominal pain, which was ultimately attributed to a splenic rupture following EMR performed near the splenic flexure of the transverse colon. This case highlights the necessity for awareness and monitoring for such uncommon but severe complications in the post-EMR period.

## 2. Timeline and narrative

### 2.1. Initial presentation and diagnosis

A 66-year-old female patient, with a history of brain contusion from a car accident and no history of abdominal surgery or chronic diseases, presented with recurrent abdominal pain. A colonoscopy revealed multiple colorectal polyps, including 3 sessile polyps approximately 0.5 × 0.5 cm in diameter near the splenic flexure of the transverse colon.

### 2.2. Procedure details

The procedure utilized an endoscope (EPK-i7000 with EC38-i10, Pentax Medical, Tokyo, Japan), a high-frequency electrosurgical unit (YHA-300, YuHua, Shandong, China), a disposable snare (SD-210U-25, Olympus Medical, Tokyo, Japan), a disposable injection needle (ATE-ZSZ-23180023*5, 5 mm, AteTec, Jiangsu, China), and a rotatable reposable soft tissue clip (ROCC-D-26-195, Nanjing Micro-Tech, Nanjing, China). During the EMR, a mixture of physiological saline, methylene blue, and epinephrine was submucosally injected at the base of the lesion using the injection needle. This caused the lesion to elevate and separate from the submucosa. The lesion’s base was then ensnared with the disposable snare, and high-frequency electrical current was applied to excise the lesion, with the tissue clip closing the wound. The specimen was sent for pathological examination. The remaining polyps were successfully removed using hot biopsy forceps. The procedure, performed under intravenous anesthesia by an experienced endoscopist, lasted approximately 10 minutes with no intraoperative abnormalities observed.

### 2.3. Immediate postoperative period

**Figure 1.**
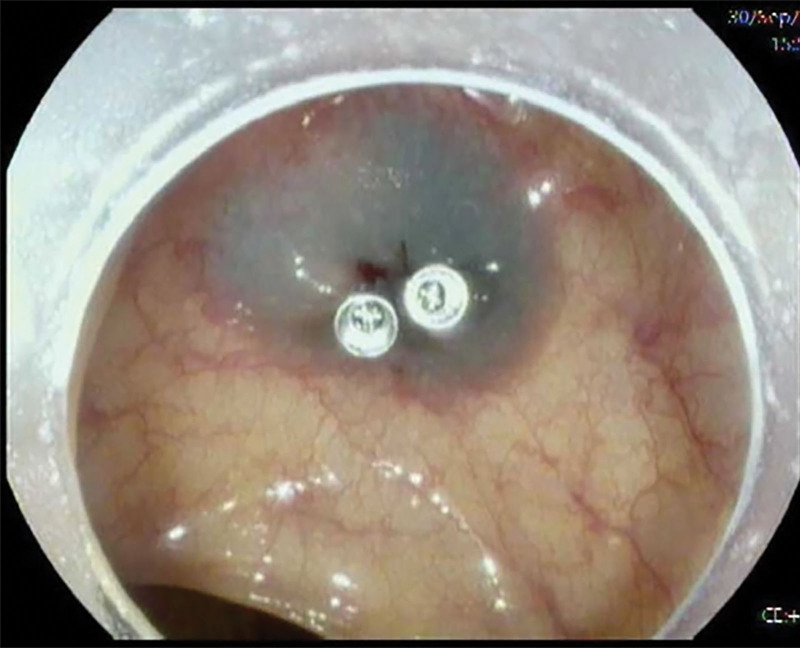
The 2nd colonoscopy showed the EMR site with the metal clip intact, and no bleeding or perforation. EMR = endoscopic mucosal resection.

Five minutes postoperatively, the patient awoke and reported lower abdominal pain that gradually worsened. Physical examination revealed mild abdominal tenderness and rebound tenderness, with stable vital signs. Considering the possibility of intestinal bleeding, perforation, and pneumoperitoneum, an emergency 2nd colonoscopy was performed. The examination showed the EMR site with the metal clip in place, submucosal blue injection liquid, and no signs of bleeding or perforation (Fig. 1). No other abnormalities were noted at the remaining surgical sites, and residual gas in the intestinal cavity was aspirated during scope withdrawal. The patient’s abdominal pain symptoms slightly improved post-procedure, but mild tenderness and rebound tenderness persisted. Given the patient’s stable vital signs and overall good condition, she was admitted to the ward for close clinical observation.

#### 2.3.1. Postoperative days 1 to 3

**Figure 2.**
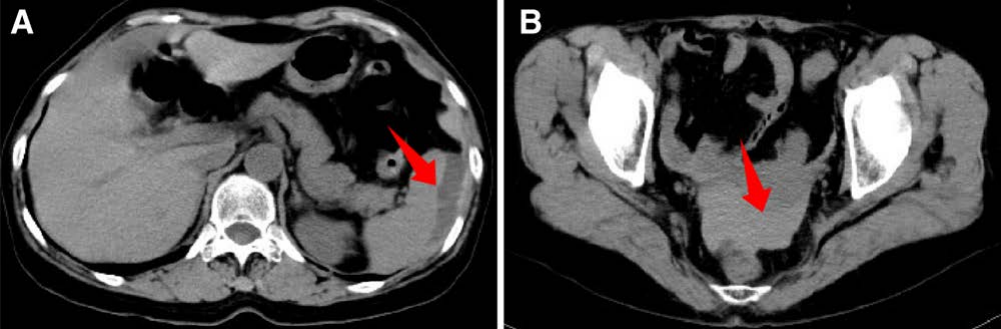
Postoperative day 3 CT images: (A) Abdominal CT shows a continuous arc-shaped low-density area under the splenic capsule (61 × 14 mm), representing a subcapsular hematoma with no signs of active bleeding (blush sign). (B) A high-density area in the rectouterine pouch (68 × 23 mm) indicates a pelvic hematoma. CT = computed tomography.

On the 3rd postoperative day, a complete blood count showed a decrease in hemoglobin from 117 g/L preoperatively to 90 g/L. An abdominal computed tomography (CT) scan indicated subcapsular splenic hemorrhage and pelvic hematocele (Fig. 2). With stable vital signs and normal coagulation function, conservative hemostasis treatment was initiated due to the absence of active bleeding.

#### 2.3.2. Postoperative days 4 to 9

**Figure 3.**
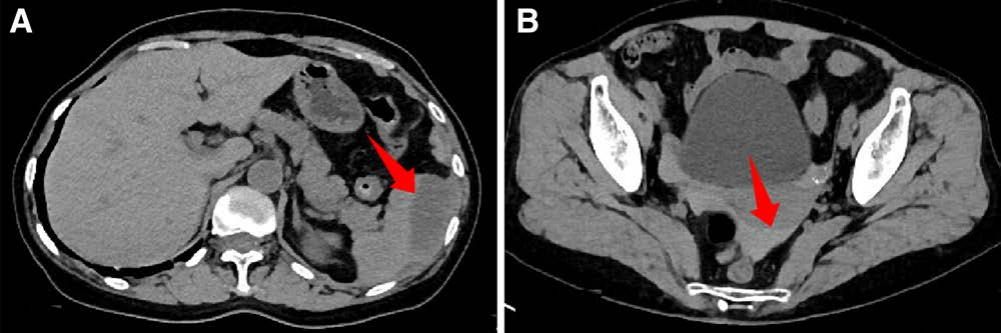
Postoperative day 9 CT images: (A) The arc-shaped low-density area under the splenic capsule has slightly increased in size (67 × 25 mm) with clear margins, representing a subcapsular hematoma. No active parenchymal bleeding is visible (blush sign). (B) The high-density area in the rectouterine pouch has slightly decreased (65 × 18 mm), indicating a pelvic hematoma. CT = computed tomography.

On the 4th postoperative day, hemoglobin increased to 93 g/L, and vital signs remained stable, continuing conservative treatment. By the 9th postoperative day, the hemoglobin level had risen to 98 g/L, and a follow-up abdominal CT scan indicated a slight increase in the size of the splenic hematoma and a slight decrease in the pelvic hematocele (Fig. 3). With stable vital signs, normal coagulation function, rising hemoglobin levels, and significantly reduced abdominal pain, the likelihood of active bleeding was considered low. Hemostatic medication was discontinued, and observation continued.

### 2.4. Discharge and follow-up

**Figure 4.**
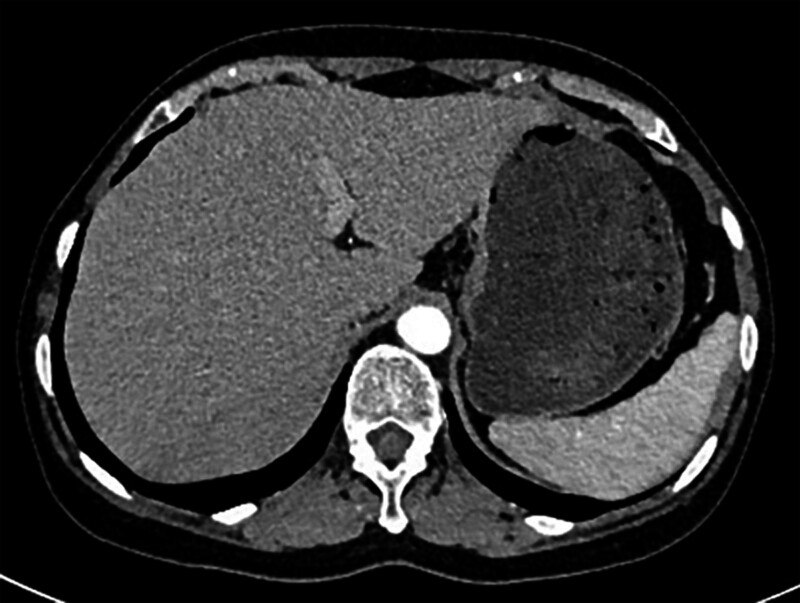
Two-year post-discharge follow-up computed tomography images show normal splenic size and morphology.

The patient was discharged on the 13th postoperative day. EMR specimen pathology indicated a mixed adenoma. No secondary infection risk, abnormal blood cell counts, splenic cysts, or splenic abscesses were observed post-discharge. A 2-year follow-up CT scan showed no significant abnormalities, with normal spleen morphology and size (Fig. 4).

## 3. Discussion

Endoscopic mucosal resection is a specialized technique for the targeted removal of lesions using colonoscopy, employing strategies such as suction, lifting, and underwater maneuvers. The procedure primarily involves the submucosal layer, making it suitable for eradicating colorectal polyps, adenomas, and incipient neoplasms.^[5]^ Regarded as a relatively safe intervention, colorectal EMR is associated with postoperative bleeding rates of approximately 2% to 24% and perforation events near 1%.^[2,6,7]^ Life-threatening incidents post-EMR, such as splenic ruptures, are exceedingly rare, and no reported cases of splenic rupture following colorectal EMR currently exist. This lack of reports may reflect the less frequent performance of EMR compared to traditional colonoscopies or a potential underdiagnosis of splenic rupture due to limited diagnostic experience, leading to minor instances being missed.

The timely monitoring of a patient’s blood routine and abdominal symptoms was pivotal in detecting a splenic rupture following an EMR in our case. A CT scan, performed in response to the development of abdominal pain after a secondary colonoscopy revealed no complications, was crucial in diagnosing the splenic rupture. This case underscores the importance for endoscopists to vigilantly monitor patients’ vital signs, blood counts, electrolytes, coagulation profiles, and other laboratory parameters perioperatively, and to remain aware of the potential for splenic rupture as an adverse event associated with EMR.

It is also imperative to recognize that EMR is contingent upon colonoscopic examination, and the inherent risk of splenic injury associated with colonoscopy should not be underestimated. Statistical data indicate that the incidence of splenic rupture following colonoscopy ranges from 0.00005% to 0.017%, with a mortality rate of up to 5.4%.^[8]^ Studies have identified several risk factors for splenic injury induced by colonoscopy, including female gender, history of smoking, prior abdominal surgery, existing splenomegaly, inflammatory bowel disease, ongoing anticoagulation therapy, multiple colonoscopic procedures, and the performance of therapeutic interventions during the procedure.^[9,10]^

**Figure 5.**
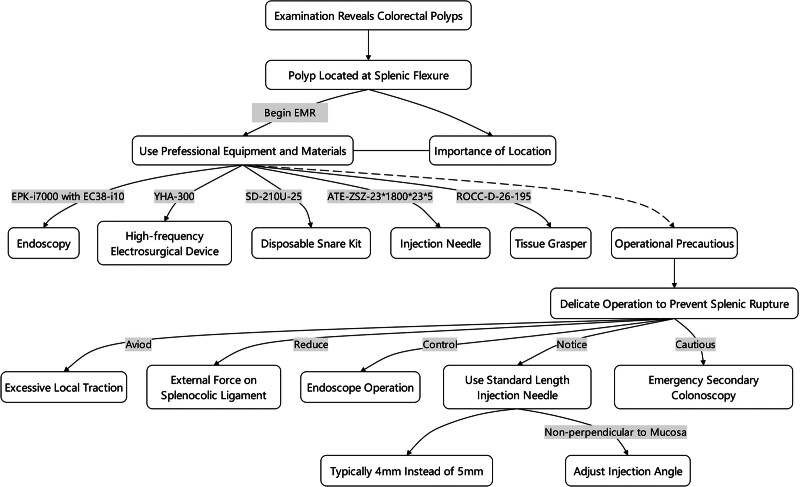
Flowchart of surgical procedures and considerations for splenic rupture.

In evaluating the splenic rupture encountered in this patient following EMR, we identified 6 contributory factors: (1) Demographics: the patient was an elderly woman, and female sex is a known risk factor for splenic rupture following colonoscopy. This risk is compounded by the patient’s advanced age and the inherent fragility of splenic tissue. (2) Medical history: the patient had sustained injuries from a vehicular accident 3 years earlier, which included a brain contusion, laceration, and intracranial hemorrhage. Despite no recorded injury to the spleen at that time, the possibility of latent damage to the spleen or surrounding structures should not be overlooked. (3) Anatomical considerations: the EMR was performed near the splenic flexure in the distal transverse colon, where local traction could potentially harm the spleen, necessitating gentle procedural maneuvers. (4) Equipment: the injection needle used during the surgery was longer than what is typically employed (5 mm as opposed to the standard 4 mm), which may pose an additional risk. (5) Procedural technique: the needle was inserted perpendicular to the mucosal surface, elevating the risk of unintentionally deep penetration and consequent splenic injury. Employing a more oblique angle upon insertion might reduce this risk. (6) Follow-up procedures: the exacerbation of the patient’s postoperative abdominal pain led to a follow-up colonoscopy to exclude severe complications such as hemorrhage or perforation. Although colonoscopy is commonly performed and considered a routine endoscopic procedure, it remains inherently invasive with associated risks such as bleeding, perforation, and traction injuries to intra-abdominal organs. The necessity of an immediate secondary colonoscopy to investigate potential complications arising from EMR deserves further discussion and scrutiny (Fig. 5). Each of these factors contributes to a complex risk profile that necessitates careful consideration in the management and follow-up of patients undergoing EMR.

We conducted a review and analysis of potential extra-intestinal organ injuries following EMR (Table 1). In our study, we identified 2 cases of post-EMR injuries: One patient experienced intra-abdominal bleeding, and another developed a retroperitoneal hematoma. The intra-abdominal bleeding likely resulted from blood accumulation in the intestinal wall due to a metal clip used to close the wound post-surgery, eventually forming an intestinal wall hematoma and rupture. The retroperitoneal hematoma was possibly caused by a tear in the mesenteric vein during surgery. Both patients initially presented with abdominal pain. As there was no active bleeding, conservative treatment was chosen, and both patients were eventually discharged after recovery. Additionally, 1 patient experienced an ovarian cyst rupture post-EMR, likely due to increased intra-abdominal pressure during the procedure. The patient presented with abdominal pain, which was alleviated with conservative treatment, and was discharged after improvement. Another patient developed left-sided pneumothorax post-surgery, with physical examination revealing diminished breath sounds on the left side. This might have been due to diaphragm damage, as the surgical area was near the gastric fundus and diaphragm. Abdominal pain was the most common initial symptom in these rare cases of extra-intestinal organ injury.

**Table 1:** A review of reported cases of rare extra-intestinal complications following endoscopic mucosal resection.

Reference	Age	Gender	Special medical history	Intervention	Surgical site	Lesion size	Main postoperative symptoms	Onset of symptoms (postoperative)	Complication	Reasons for consideration	Treatment	Hospitalization days	Prognosis
Yoshinori et al^[11]^	84	F	AF (oral rivaroxaban 30 mg/day)	EMR	Hepatic flexure	0.5 cm	Abdominal pain	Day 1	Intra-abdominal bleeding	Intramural intestinal hematoma rupture	Conservative treatment	8 days	Improved and discharged
Reo et al^[4]^	57	M	–	EMR	–	–	Abdominal pain	Day 0	Retroperitoneal hematoma	mesenteric vein tear	Conservative treatment	43 days	Improved and discharged
Redha et al^[12]^	60	F	Ovarian cyst	EMR	Transverse colon	1.5 cm	Abdominal pain	Day 0	Ovarian cycle rupture; non-perforated pneumoperitoneum	Increased intra-abdominal pressure	Conservative treatment	7 days	Improved and discharged
Myeongseok et al^[13]^	45	F	–	EMR	Gastric fundus	1 cm	Left respiratory sound weakened	Day 0	Left pneumothorax	Perforation of diaphragm	Conservative treatment	5 days	Improved and discharged
Present case	66	M	–	EMR	Splenic flexure	0.5 cm	Abdominal pain	Day 3	Splenic rupture	Six factors, see discussion section	Conservative treatment	13 days	Improved and discharged

AF = atrial fibrillation, EMR = endoscopic mucosal resection, F = female, M = male.

We further reviewed literature on splenic injuries following colonoscopy or related treatments^[14–20]^ (Table 2). The results indicated that the majority of patients with splenic rupture initially presented with abdominal pain, including upper left abdominal pain, left shoulder pain (Kehr sign), and lower abdominal pain. A few patients exhibited symptoms such as dizziness, syncope, and unstable vital signs. Diagnosis primarily relied on CT scans, and treatment varied based on the patient’s specific conditions, including conservative treatment, splenic artery embolization, laparoscopic splenectomy, and open splenectomy. The choice of treatment should consider the patient’s hemodynamic status, degree of splenic injury, underlying diseases, and comorbidities. According to the American Association for the Surgery of Trauma classification system, splenic injuries are categorized into 5 grades (I–V) based on severity. Grade I includes lacerations <1 cm in depth and subcapsular hematomas involving <10% of the surface area. Grade II comprises lacerations measuring 1 to 3 cm in depth and subcapsular hematomas covering 10% to 50% of the surface area. Grade III involves lacerations >3 cm and subcapsular hematomas involving more than 50% of the surface area or expanding. Grade IV is characterized by hilar injuries, and grade V refers to a shattered spleen.^[18,21]^ For hemodynamically stable patients with confined intraparenchymal bleeding (grade III and below), conservative treatment can be considered. This approach can avoid surgery-related complications and maintain the patient’s structural integrity.^[22]^ However, statistics show that approximately 70% of patients with colonoscopy-related splenic injuries eventually underwent splenectomy.^[9]^ Therefore, early detection and timely management are crucial for patients with splenic rupture post-colonoscopy or treatment. Abdominal pain is a common complication post-colonoscopy or treatment and should not be taken lightly, especially if persistent or worsening. Vital signs should be promptly monitored, and an abdominal CT scan should be conducted. Early suspicion and detection are key to ensuring patients receive timely and optimal treatment. In this case, due to timely detection, mild injury (grade II), and hemodynamic stability, the patient successfully underwent active conservative treatment with hemostatic agents.

**Table 2:** A review of selected cases of splenic rupture following colonoscopy or related treatments.

Reference	Age	Gender	Special medical history	Intervention	Main postoperative symptoms	Onset of symptoms	Complication	Diagnosis	AAST	Vital signs	Time to diagnosis	Treatment	Hospitalization days	Prognosis
Diego et al^[14]^	60	F	Abdominal surgery	Colonoscopy/polypectomy	Abdominal pain	Day 1	Splenic rupture	CT	–	Stability	Day 4	Conservative treatment	–	Improved and discharged
Marco et al^[15]^	73	M	–	Colonoscopy/polypectomy	Abdominal pain	Day 2	Splenic rupture	CT	III	Instability	Day 2	Surgical treatment	6 days	Improved and discharged
Nehal et al^[16]^	75	F	–	Colonoscopy	Abdominal pain/left shoulder pain	Day 0	Splenic rupture	CT	III	Stability	Day 0	Conservative treatment	6 days	Improved and discharged
Guerra et al^[17]^	60	F	–	Colonoscopy/polypectomy	Diffuse abdominal pain and distension	Day 0	Splenic rupture	CT	–	Instability	Day 0	Surgical treatment	4 days	Improved and discharged
Sandra et al^[18]^	73	F	–	Colonoscopy	Abdominal pain/syncope	Day 0	Splenic rupture	CT	–	Instability	Day 0	Surgical treatment	13 days	Improved and discharged
Steven et al^[19]^	59	F	Abdominal surgery	Colonoscopy	Abdominal pain	Day 0	Splenic rupture	CT	II–III	Stability	Day 0	Conservative treatment	10 days	Improved and discharged
Beatriz et al^[20]^	40	F	Abdominal surgery	Colonoscopy/polypectomy	Abdominal pain	Day 1	Splenic rupture	CT	–	Stability	Day 1	Conservative treatment	–	Improved and discharged
Beatriz et al^[20]^	80	M	Anticoagulants	Colonoscopy/polypectomy	Abdominal pain/dizziness	Day 1	Splenic rupture	CT	–	Instability	Day 1	Surgical treatment	–	Improved and discharged
Alberto et al^[20]^	65	F	–	ESD	Dizziness/hypotension	Day 1	Splenic rupture/large hemoperitoneum	CT	–	Instability	Day 1	Surgical treatment	6 days	Improved and discharged
Present case	66	F	–	EMR	Abdominal pain	Day 0	Splenic rupture	CT	II	Stability	Day 3	Conservative treatment	13 days	Improved and discharged

American Association for the Surgery of Trauma Splenic Injury Scale.

AF = atrial fibrillation, AAST = the American Association for the Surgery of Trauma, CT = computed tomography, EMR = endoscopic mucosal resection, ESD = endoscopic submucosal dissection, ESD = endoscopic submucosal dissection, F = female, M = male.

The free blood sign is a common indicator of splenic rupture, referring to atypical color changes observed through the intestinal wall due to intra-abdominal blood.^[23]^ During colonoscopy, a blue discoloration of the intestinal wall can typically be observed. The shape of the free blood sign varies with the patient’s position changes. In our 2nd colonoscopy, the blue sign at the EMR site did not change shape, likely due to the injection of methylene blue solution during surgery.

To prevent and screen for the risk of splenic rupture following colonoscopy and related treatments such as EMR, a comprehensive preoperative history assessment and imaging examination should be conducted, with particular attention to any history of abdominal surgery, trauma, or splenomegaly. During the procedure, appropriate instruments should be selected, and adjustments made to the needle angle and force to avoid the use of excessively long needles and vertical punctures, maintaining gentle movements throughout. Additionally, caution should be exercised when considering a 2nd colonoscopic examination; alternative imaging examinations may be considered if postoperative complications are suspected. Postoperatively, symptoms should be closely monitored, high-risk patients should be regularly followed up, and complications should be detected early. Patients should be thoroughly informed about the preoperative and postoperative risks and precautions, and guided to pay attention to any bodily changes and seek medical advice promptly. These measures can effectively reduce the incidence of splenic rupture and ensure the safety of high-risk patients.

## 4. Conclusion

Although splenic rupture after EMR is extremely rare, it is a serious and potentially life-threatening complication. When obtaining informed consent, it is important to emphasize not only common complications like bleeding and perforation but also the risk of splenic injury. Physicians should select appropriate instruments and carefully adjust the angle and force of needle insertion, avoiding excessively long needles and vertical insertion. The procedure should be performed gently to minimize the risk of splenic rupture. For lesions near the splenic flexure, if postoperative abdominal pain occurs, regardless of left shoulder pain, splenic rupture should be considered, and a CT scan promptly performed. Postoperatively, physicians should closely monitor vital signs and repeatedly check blood counts and coagulation parameters. Treatment should be tailored to the splenic injury’s extent and the patient’s overall condition, with immediate surgery if necessary. High-risk patients should be regularly followed up and instructed to monitor for physical changes. Endoscopists should remain vigilant during procedures, fully understanding potential complications and closely monitoring the patient’s condition postoperatively. This vigilance is key to preventing severe complications and ensuring optimal outcomes.

## Author contributions

**Conceptualization:** Yusong Ye, Rui Yang, Weixing Yang.

**Data curation:** Yusong Ye, Rui Yang, Weixing Yang.

**Formal analysis:** Yusong Ye, Rui Yang, Qilang Xiang.

**Investigation:** Rui Yang.

**Methodology:** Yusong Ye, Rui Yang.

**Project administration:** Yusong Ye.

**Resources:** Yusong Ye.

**Supervision:** Yuexi Chen, Weixing Yang, Muhan Lü.

**Visualization:** Muhan Lü.

**Writing – original draft:** Yusong Ye, Rui Yang, Shicheng Peng, Yuexi Chen.

**Writing – review & editing:** Weixing Yang, Muhan Lü.
